# How Moving Together Brings Us Together: When Coordinated Rhythmic Movement Affects Cooperation

**DOI:** 10.3389/fpsyg.2016.01983

**Published:** 2016-12-22

**Authors:** Liam Cross, Andrew D. Wilson, Sabrina Golonka

**Affiliations:** ^1^Department of Psychology, Lancaster UniversityLancaster, UK; ^2^Psychology, School of Social Sciences, Leeds Beckett UniversityLeeds, UK

**Keywords:** coordinated rhythmic movement, interpersonal entrainment, interpersonal synchrony, interpersonal coordination, rhythmic entrainment, joint action, social cognition, cooperation

## Abstract

Although it is well established that rhythmically coordinating with a social partner can increase cooperation, it is as yet unclear when and why intentional coordination has such effects. We distinguish three dimensions along which explanations might vary. First, pro-social effects might require in-phase synchrony or simply coordination. Second, the effects of rhythmic movements on cooperation might be direct or mediated by an intervening variable. Third, the pro-social effects might occur in proportion to the quality of the coordination, or occur once some threshold amount of coordination has occurred. We report an experiment and two follow-ups which sought to identify which classes of models are required to account for the positive effects of coordinated rhythmic movement on cooperation. Across the studies, we found evidence (1) that coordination, and not just synchrony, can have pro-social consequences (so long as the social nature of the task is perceived), (2) that the effects of intentional coordination are direct, not mediated, and (3) that the degree of the coordination did not predict the degree of cooperation. The fact of inter-personal coordination (moving together in time and in a social context) is all that's required for pro-social effects. We suggest that future research should use the kind of carefully controllable experimental task used here to continue to develop explanations for when and why coordination affects pro-social behaviors.

## Introduction

It is well-established that moving in time with other people can increase cooperation between co-actors (Anshel and Kipper, 1988; Wiltermuth and Heath, 2009; Kirschner and Tomasello, 2010; Reddish et al., 2013, 2014; but see Kirschner and Ilari, 2014), though, it is still unclear what it is about these *Coordinated Rhythmic Movement* (CRM) tasks that makes people more cooperative. Previous work has identified a number of interesting effects and it is now time to begin trying to explain why these effects occur. At present, this work is complicated by the sheer variety of paradigms employed to generate and measure these effects. The purpose of this paper is to try to lay the groundwork for developing an explanation of the pro-social effects of coordination. We do this by tackling a number of basic questions about the effect using a single, well-understood, CRM paradigm.

In this paper we consider some classes of model that could characterize how coordination impacts cooperation. These models vary along three dimensions: (1) whether increased cooperation depends on in-phase *synchrony* (S+) or *coordination*, more generally (S−), (2) whether the relationship between social coordination and cooperation is *direct* (D+) or *mediated* (D−), and (3) whether cooperation varies *in proportion* to coordination at the individual level (P+), or whether there is a *threshold* effect (P−). The first dimension relates to whether synchronous (in-phase) movements are necessary to impact cooperation, or whether other coordinations (e.g., anti-phase) can also affect cooperation. The second dimension concerns whether there is a direct path between social coordination and cooperation, or whether this relationship is mediated by other factors, such as group cohesion (e.g., Wiltermuth and Heath, 2009). The third dimension concerns whether there is a linear relationship between coordination and cooperation at the level of individual participants, or whether pro-social benefits obtain (and then remain more or less constant) after a certain threshold in coordination is reached. These models are, themselves, descriptive rather than explanatory. However, this work moves us further down the road toward explanation by explicitly identifying the features that any future explanatory model must possess. We first discuss the dimensions of interest in more detail below with reference to the existing evidence from the literature in favor of particular classes of models. We then summarize our choice of movement task and explain how it enables us to test the dimensions of interest, thereby helping us home in on essential features that an explanatory model of the pro-social effects of intentional coordination must possess.

## In-phase synchrony vs. coordination (S+ vs. S−)

Movements are *coordinated* when two rhythmically moving limbs (oscillators) move so as to maintain some relative phase with respect to one another. Movements are *synchronous* when those limbs move in-phase (i.e., at 0° relative phase). During in-phase movements, the two oscillators move in the same direction at the same time. During anti-phase (180° relative phase) movements, each oscillator moves in the opposite direction as its partner at the same time. Throughout this work the term synchrony is used to refer to in-phase movements only (in line with the general literature on coordination, e.g., Kelso, 1995), although elsewhere anti-phase has sometimes been treated as an example of synchrony (e.g., Miles et al., 2010). Our definition of synchrony was chosen in order to allow us to easily discriminate between strict in-phase synchronization and other forms of coordination (i.e., anti-phase). Technically, successfully moving so as to maintain *any* relative phase (from 0 to 360°) is an instance of a coordinated rhythmic movement (although there are well known limits to the coordinations humans can produce without extensive training; Kelso, 1995). The question is whether the pro-social effects of coordination reported in the literature, are actually restricted to cases where the coordination is in-phase (synchronous movements).

If coordination, generally, and not just in-phase synchrony, has positive consequences on cooperation, then the effects should be obtained following coordination at any relative phase. We currently lack evidence to support this idea because the majority of tasks used to test the pro-social effects of coordination rely exclusively on in-phase coordination (and, to our knowledge, our experiment is the first work to address the effects of anti-phase coordination on cooperation, specifically). Those that have employed anti-phase conditions have found mixed evidence concerning whether anything besides in-phase synchrony impacts social variables (e.g., Miles et al., 2010; Cirelli et al., 2014; Sullivan et al., 2014). To begin disambiguating the effects of in-phase synchrony from the effects of coordination more generally, Experiment 1 explicitly compares the effects of in-phase and anti-phase coordination on post-task cooperation.

## Direct vs. indirect effect (D+ vs. D−)

The effect of coordination on pro-social variables is indirect if coordination must impact an intervening variable (e.g., group cohesion) or coincide with a causally relevant variable (e.g., social context) in order to affect cooperation. If this is the case, then coordination only has positive consequences for pro-social variables by virtue of its effect on something like group cohesion or by providing the opportunity to engage in a certain type of social context. In contrast, the effect of coordination on pro-social variables could be direct. If the relationship is direct then coordination would not need to impact an intervening variable or coincide with another causally relevant variable to influence cooperation.

The literature, to date, is conflicted concerning directness. We first consider evidence for a mediating variable between coordination and cooperation. Research has focused exclusively on two potential mediators—group cohesion and self-other-overlap. Group cohesion is the feeling of being on the same team and being emotionally connected with other group members. Wiltermuth and Heath (2009), Wiltermuth (2012) found that levels of post-task group cohesion were related to the social effects of coordination, though others (e.g., Reddish et al., 2013; Lumsden et al., 2014; Dong et al., 2015) found no such relationship. The discrepancy in results may be, at least, partially explained by differences in how group cohesion was conceptualized and measured. Reddish et al. (2013) grouped emotional connection, trust and self/other overlap (the extent of self-rated overlap between oneself and others) into a single construct, which was termed group cohesion, after factor analysis suggested they all tap a similar construct. Wiltermuth (2012), on the other hand, measured group integrators only (i.e., perceived closeness, connectedness and similarity to the group) and labeled the construct emotional connection (see also Wiltermuth and Heath, 2009; Lumsden et al., 2014; Dong et al., 2015).

Others have investigated self-other-overlap as a potential mediator of the relationship between coordination and cooperation; again, evidence for the mediated model is inconclusive. Lumsden et al. (2014) and Reddish et al. (2013) found evidence in favor of a mediating relationship, while Reddish et al. (2014) found no evidence for such a relationship. As before, it is difficult to draw conclusions from the literature given the plurality of methods and measures.

Another way the effect of coordination on pro-social variables could be thought of as in/direct depends on whether a coordination task, in and of itself, (i.e., absent a particular social context), is sufficient to impact coordination. If it is direct in this way then coordinating movements with, say, a metronome or a computer display rather than a co-actor, would be sufficient to lead to social consequences. If it is indirect in this way, then coordination must be accompanied by some kind of social context to impact pro-sociality; i.e., effect would not be due to coordination “*per se*”—coordination *itself* and/or coordination *by itself*. There is considerable evidence that some kind of social context is an important element to obtain positive social effects following coordination tasks (Hove and Risen, 2009; Kirschner and Tomasello, 2009; Wu et al., 2013; Launay et al., 2014), however, questions remain about how much social context is necessary and whether this relationship is one of mediation or moderation.

In sum, the evidence from previous research is inconclusive about whether coordination must impact an intervening variable in order to have positive consequences on cooperation. Evidence is stronger for the idea that coordination must coincide with a social context in order to affect cooperation. The studies reported below provide the strongest evidence to date for D+ vs. D− models by testing a variety of potential mediators (i.e., group cohesion, self-other overlap, trust, self-rated success at coordination, self-rated task difficulty, task difficulty, and mood) within subjects at both pre- and post-coordination. In line with the substantial existing evidence that social context is important, all of the studies below involve pairs of participants completing an intentional coordination task together; however Followup 1 manipulates whether the information participants use to coordinate is social or non-social.

## Individual vs. group level effects (P+ vs. P−)

Whether the effect of intentional coordination on cooperation is direct or indirect, there are two main types of relationship we might observe between these variables. The first possibility is that individual measures of coordination success predict individual levels of cooperation. That is, changes in cooperation occur in proportion to changes in coordination success. The second possibility is that there is a threshold relationship between coordination and cooperation. In this case, coordination would positively influence cooperation as long as some minimum threshold of coordination success was achieved.

Previous research paints a mixed picture in terms of what to expect on this dimension. The only work focusing on cooperation to take actual measures of coordination found that coordination did not predict cooperation (Kirschner and Ilari, 2014), but this result is limited by the fact that they found no effect of coordination on cooperation anyway. Looking beyond cooperation to other social variables does little to clarify the picture. On the one hand, there is evidence that tightness in movement coupling predicts likability between co-actors (Hove and Risen, 2009). On the other hand, coordination success is not a good predictor of post-task trust (Launay et al., 2013). The studies reported below compare P+ vs. P− models by testing whether individual level success at coordination predicts subsequent individual level cooperation behavior.

### Our coordination task

Researchers have used a variety of tasks to investigate the effect of intentional coordination on pro-sociality (e.g., waving cups and singing: Wiltermuth and Heath, 2009; flexing and extending arms: Miles et al., 2010). It is difficult to lay the groundwork for an explanatory model using results from such a variety of complex tasks. It would be preferable to identify a coordination task that is simple enough to study but that is complex enough to allow all the necessary manipulations required to investigate when and how coordination affects social behavior. We believe we have found such a task and this is described below, though, first we explain in more detail the basic structure of CRM tasks, generally.

CRM tasks are essentially perception-action tasks, and have typically been studied as such in the experimental literature (e.g., Kelso, 1995; Bingham, 2001, 2004). They involve the continuous control and matching of rhythmic movements via perceptual information about the coordination between those movements. The rhythm of a CRM is defined by the relative phase between the oscillating movements. Movements are coordinated when a particular relative phase is maintained within some error band. As discussed earlier, in-phase coordination occurs when the movements are in the same direction at the same time, while anti-phase coordination occurs when the movements are in the opposite direction at the same time. The remaining range of coordinated movements is generally described as “out-of-phase.” The basic phenomena of a CRM task are that movements are stable at in- and anti-phase, while movements at any other phase are difficult to maintain and highly variable. In-phase movements are more stable than anti-phase movements and, if the frequency of anti-phase movements is increased to around 3–4 Hz they transition to in-phase. These effects persist when the coordination is enacted between two people (Schmidt et al., 1990) and between a person and a point light display (e.g., Wilson et al., 2005a,b). This indicates that the ability to maintain rhythmic coordination depends on a perceptual coupling of information specifying relative phase between oscillators.

Bingham et al. (Bingham, 2001, 2004; Snapp-Childs et al., 2011) have developed a model of CRM (the Bingham model) using a task where participants move joysticks from side to side at some relative phase to coordinate the motions of two dots on a computer screen. The screen shows a point light display representing the limbs' motions (see also Wilson et al., 2005a,b). This task contains all the critical elements of a CRM task: voluntary control of limbs, coordination of limbs with a co-actor and perceptual control of the coordination. The Bingham model explains the above phenomena by explicitly modeling the perception-action components involved in the task. Several papers have empirically validated the main predictions of the model (Wilson and Bingham, 2008; Wilson et al., 2010; Snapp-Childs et al., 2011).

The studies below are based on the task used by Bingham and colleagues to develop an explanatory model of CRM. This CRM task is particularly well-suited to the job of discriminating S+ and S− models, as it's possible to run the task with any target relative phase, and of discriminating P+ and P− models, as it allows us to compute precise and sensitive measures of coordination that can be used to determine how much actual coordination predicts post-task measures, if at all. We can then combine data from this task and other measures to discriminate D+ and D− models as well.

This choice of task is also ideal for constructing an appropriate control task, which has proven a major challenge in the literature. A good control task must be comparable to the CRM task, involving co-actors making comparable movements (though ones that are not rhythmically coordinated with their co-actors). However, control tasks in the six papers looking at how CRM affects cooperation varied considerably in how closely they match the experimental task (see Table 1). Some previous work has even used anti-phase movements as a control condition. However, as noted above, moving anti-phase (or even out-of-phase) with someone is still a type of CRM. People can and do entrain at anti-phase, and similar social effects might also be fostered by anti-phase interpersonal entrainment (see Cirelli et al., 2014). Tasks involving completely disparate activities such as doing a jigsaw (Reddish et al., 2014) or watching a documentary (Anshel and Kipper, 1988) may also not be appropriate controls, as they are too different from the experimental tasks at hand. For example, tapping one's foot in time to a metronome with two other people is not very similar to doing a jigsaw with two other people (Reddish et al., 2014), as these tasks vary in multiple ways (i.e., one includes music and one does not, one includes coordinating your moments with the other person in a certain way while one does not employ movement coordination at all). This makes interpreting findings between conditions as the result of CRM difficult, if not impossible.

**Table 1:** **Experimental and control tasks used in studies looking at CRM's effects on cooperation**.

	**Entrainment task**	**Control task**
Anshel and Kipper, 1988	Group singing	Listing to music/watching a documentary
Wiltermuth and Heath, 2009, Exp 1	Synchronized walking	Walking normally
Wiltermuth and Heath, 2009, Exp 2 and 3	Synchronous cup waving and singing in time to Canadian anthem	Static cup holding and silently reading lyrics while listening to Canadian national anthem
Kirschner and Tomasello, 2010	A game involving synchronously singing and walking in time to music	A game involving walking and vocalizing non-synchronously with no music
Reddish et al., 2013, Exp 1	Synchronous movements in time to a metronome	Watching a video of other people performing the task
Reddish et al., 2013, Exp 3	Synchronized foot tapping	Asynchronous foot tapping
Kirschner and Ilari, 2014	Synchronized drumming	Solitary drumming
Reddish et al., 2014, Exp 2	Synchronized foot tapping	Completing a jigsaw puzzle

Our CRM task is amenable to a straight forward, well-matched control task whereby participants are instructed to move their joysticks at different frequencies while performing different movements. This control condition is minimally different from coordinated conditions (both involve rhythmically moving a joystick at a specified frequency), while breaking the coordination between partners.

### The current studies

The goal of the studies that follow is to begin homing in on the class of model that best captures the relationship between intentional coordination and cooperation. This work will place specific, empirically-driven constraints on future work concerning the mechanism by which coordination influences cooperation. Experiment 1 was designed to discriminate between S+ and S− models (in-phase synchrony or coordination), between D+ and D− models (direct or mediated), and between P+ and P− models (group or individual level effect). Based on the results of this experiment we conducted two follow-ups. The first further explores the S+/S− distinction by investigating the consequences of coordinating via social and non-social information. The second probes the necessary features of a coordination task by testing two control tasks.

## Experiment 1

Experiment 1 tested whether in-phase synchrony is necessary to the effect of coordination on cooperation or whether the effect obtains with other coordinations as well (S+ or S−). Since our task allows a kinematic record of each participant's movements, we also tested whether cooperation varies in proportion to coordination, allowing us to discriminate between P+ and P− models. Finally, we measured several potential mediators suggested from previous research, which provides some evidence for D+ vs. D− models.

## METHODs

### Participants

Sixty-six undergraduate students at Leeds Beckett University volunteered to participate (19 males and 47 females *M*_age_ = 19.17 year, *SD*_age_ = 2.77). All participants were naive to the aims of the study. The experiment was approved by the Leeds Beckett University Psychology Ethics Review Board.

### Design

The study employed an experimental design with one between-subjects factor: Movement Phase. This had three levels: in-phase (0°), anti-phase (180°), or no coordination (control).

### Tasks and measures

#### Movement

In both experimental conditions, pairs of participants, sitting side by side moved one joystick each (Logitech Pro joysticks with force feedback disabled) horizontally at 0.75 Hz using a point light display (PLD) to monitor their and their partner's movements. The PLD consisted of two white feedback dots displayed on a black background by a single laptop screen positioned approximately 1 m in front of them. The dots were 40 × 40 pixels, and separated by a visual angle of 0.14°, one above the other, positioned in the center of the screen (Wilson et al., 2005a,b, 2010; Snapp-Childs et al., 2011). In the in-phase condition, participants moved so as to maintain 0° relative motion between their and their partner's dots. In the anti-phase condition, participants moved so as to maintain 180° relative motion between their and their partner's dots.

For the control task, participants made uncoordinated movements at different frequencies. One participant always moved their joystick at 0.6 Hz and the other always moved at 0.9 Hz (0.75 ± 0.15 Hz). Participants alternated moving their joysticks vertically and in clockwise circles, so that partners never performed the same movement during a trial. Participants switched movements every trial (e.g., person 1 moved vertically on one trial, in circles on the next etc.; person 2 in the pair did the opposite).

Participants in all conditions first saw two 15 s demonstrations of dots moving at the desired phase and frequency. In the experimental conditions both dots moved at 0.75 Hz (at either 0 or 180° relative to each other). In the control condition one dot moved at 0.6 Hz and the other at 0.9 Hz. After each demo participants had 30 s practice time to acquaint themselves with the required movements. Following this brief initial practice, participants completed six 60 s trials. Each trial was preceded by a four second version of the demonstration pacing them to the required phase and frequency of movements. This experiment was run on a MacBook Pro with a custom Matlab toolbox programmed by the second author and incorporating the Psychtoolbox (Brainard, 1997; Pelli, 1997; Kleiner et al., 2007).

#### Social mediators

##### Self/other overlap

Self/other overlap was measured using the Inclusion of the Other in Self (IOS) scale (Aron et al., 1992). Participants were asked to indicate how much overlap they felt between themselves and the other participant by choosing from one of seven different diagrams. The diagrams consist of increasingly overlapping circles, one representing the self and one representing the other (see Data Sheet 1).

##### Cohesion scale

Five questions were used to measure mood, trust and cohesion (see Data Sheet 2). Question 1 measured participants' mood. Question 5 measured how much participants trusted each other. Questions 2–4 measured participants' cohesion to each other (closeness, connectedness and similarity). These were the same questions as have previously been used to measure cohesion in Wiltermuth and Heath (2009). Participants recorded their responses to each of these questions by marking a 185 mm continuum. This response scale was used to make it more likely to detect any changes after the movement manipulation and has been successfully used in a similar context by Lumsden et al. (2014).

#### Dependent variables

##### Economic game

This included both a Public Goods Game (PGG) and an investment game (see Data Sheet 3). The PGG was identical to that used by Wiltermuth and Heath (2009) except token values were changed from dollar amounts to points. Participants were given a response booklet containing instructions and response sheets for each of five rounds of play. The aim of the game was to collect as many points as possible. In order to encourage competition between participants, the person who collected the most points won £40 of vouchers. For each of the five rounds participants had ten tokens to allocate between two accounts, a private account and a public account. Each token in the public account was worth three points to each of the players, while each token in the private account was worth five points only to the player who allocated that token. In each round participants privately recorded how many tokens they wished to allocate to each of the two accounts.

##### Investment game

After Round 5 of the PGG, participants played an investment game (adapted from Berg et al., 1995) to measure trust and reciprocity. Participants had the chance to transfer/invest the points (none, a quarter, half, or all) that they had earned in the public goods game. Any points that were invested were automatically doubled but it was up to the other player how many of these points to return to them (none, only the original amount invested, the original investment plus half of the earned bonus, or all of the original investment and the earned bonus). Each participant acted as both investor and banker simultaneously by confidentially marking their choices on a separate sheet without any discussion.

### Procedure

This study was conducted in pairs. Sessions lasted approximately 25 min. Participants completed the IOS and the cohesion scale (pre-test measures of potential mediators, and mood item) followed by the movement task. Participants then rated their perceived success at the coordination task as well as task difficulty and enjoyment using four-point Likert scales. Next, participants completed a second copy of the IOS and cohesion scale (post-test measures of potential mediators, and mood item). Finally, participants took part in the Economic (public goods and investment) Game.

## Results

We checked whether mood, task difficulty, task enjoyment, and perceived success differed between in-phase, anti-phase, and control tasks. The distribution of scores on each of these variables was found non-normal from Shapiro-Wilkes tests (SW tests of normality used throughout) (*p*'s < 0.05). Kruskal-Wallis tests confirmed that scores on these variables did not differ between movement tasks (all *p*'s > 0.05). It was therefore concluded that mood, task enjoyment, perceived task difficulty or perceived success did not contribute to the effects described below.

### Coordination

All movement trials except for the first two practice rounds were analyzed. A low-pass Butterworth filter with a cut-off frequency of 10 Hz filtered each dot's position time series. A 60 Hz time series of the relative phase between the two dots was computed as the difference between the arctangent of each dot's velocity over position at each sample.

Mean vector length (MVL) is the circular equivalent of the standard deviation (Batschelet, 1981; see Wilson et al., 2005a,b for more detail). It is the normalized length of the resultant vector obtained by summing the relative phase vectors from each time step and measures coordination stability. MVL ranges from 0 (indicating minimum stability, a uniform circular distribution) to 1 (indicating maximum stability, no variability).

**Figure 1:**
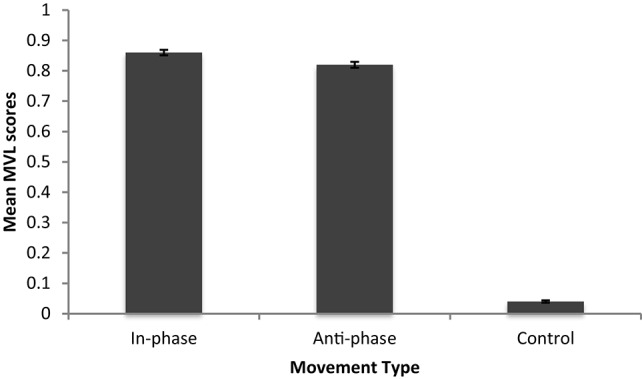
**Mean MVL scores for Experiment 1**.

The distribution of MVL scores of those who moved in-, anti-phase and those who did not coordinate all differed significantly from normality (*p*'s < 0.05). An independent samples Kruskal-Wallis test identified a significant effect of phase on coordination scores [*H*_(2)_ = 47.29, *p* < 0.001]. Bonferonni *post-hoc* tests with adjusted *p*-values (for 3 pairwise comparisons) showed more stable coordination for those moving in- and anti-phase than in the control condition (see Figure 1 for mean MVL scores), *p*'s < 0.001. Coordination at anti-phase did not significantly differ from coordination at in-phase (*p* > 0.05)^1^.

### Cooperation

**Figure 2:**
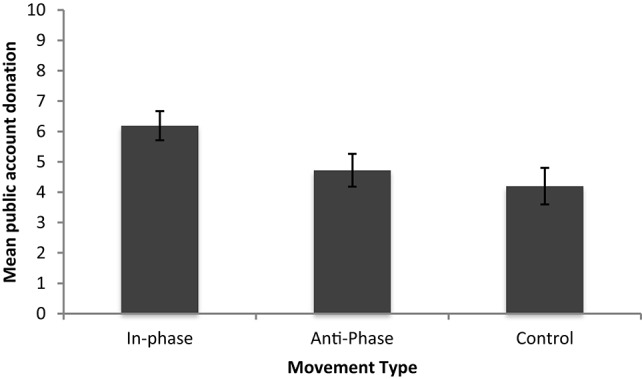
**Mean public account donations for Experiment 1**.

Next we examined whether participants in the in- and anti-phase conditions were more cooperative post movement task than those in the control condition. A univariate ANOVA found a significant effect of phase on the mean public account donation [*F*_(2, 63)_ = 3.62, *p* < 0.05, ŋ^2^ = 0.10]. Bonferroni *post-hoc* tests indicated that the only significant difference lay between those who moved in-phase and the control (*p* < 0.05), no other comparison was significant (*p*'s > 0.05). Post-coordination cooperation was greater for participants in the in-phase group compared to the control group (Figure 2).

Next we conducted a simple linear regression with each pair's MVL scores and each pair's average public goods donation to determine if the degree of coordination success predicts the degree of cooperation. A pair's coordination score did not significantly predict their average cooperation score [*F*_(1, 31)_ = 3.19, *p* > 0.05, *r*^2^ = 0.093].

### Trust and reciprocity

Trust was measured using the first part of the investment game (choosing what to invest with the other player: investing nothing, a quarter, half, or all). The distributions of those who moved in-phase, anti-phase, and those who did not coordinate all deviated significantly from normality (*p*'s < 0.05). A Kruskall-Wallis test showed no significant difference in trust between those who moved at in-, anti-phase and those who did not coordinate [*H*_(2)_ = 4.48, *p* > 0.05].

As a further check that coordination had no effect on trust, we compared self-reported measures of trust across the coordination conditions. Change scores for the self-reported trust measure were first calculated by subtracting each person's “before” score from their “after” score. The distributions for those who moved in-phase, anti-phase, and those who did not coordinate all deviated significantly from normality (*p*'s < 0.05). Consistent with the measure of trust based on the investment game, a Kruskall-Wallis test showed no significant change in self-reported trust between those who moved at in-, anti-phase, and those who did not coordinate [*H*_(2)_ = 3.87, *p* > 0.05].

Reciprocity was measured using the option chosen in the second part of the investment game (choosing to return nothing, return only the original investment, return the original investment plus half of the bonus, or, return the original investment plus all of the bonus). Reciprocity scores for those who moved in-, anti-phase and those who did not coordinate all deviated significantly from normality (*p*'s < 0.05). A Kruskall-Wallis test showed no significant difference in reciprocity between those who moved at in-, anti- phase, and those who did not coordinate [*H*_(2)_ = 4.11, *p* > 0.05].

### Potential mediators (group cohesion and self/other overlap)

Change in group cohesion was measured as the sum of the difference between the three cohesion change questions (how similar/close/connected they felt to each other). A univariate ANOVA with phase (in-, anti-phase, no coordination) showed no significant effect of phase on group cohesion [*F*_(2, 63)_ = 1, *p* > 0.05].

Change in self-other overlap was measured as the difference in self-other overlap before and after engaging in the coordination task (post-coordination—pre-manipulation). The distribution of overlap change scores for those who moved at in-, anti-phase, and those who did not coordinate all deviated significantly from normality (*p*'s < 0.05). An independent samples Kruskal-Wallis test showed no significant effect of phase on changes in overlap between the three conditions [*H*_(2)_ = 0.262, *p* > 0.05].

Analysis previously reported also confirmed that self-report measures of trust, mood, task difficulty, task enjoyment and perceived success did not differ between movement conditions.

## Discussion

### Overview

The results showed that participants who moved in-phase with one another were more cooperative than those who moved in an uncoordinated manner. None of the measured candidate mediators were related to cooperation, and cooperation was not predicted by the level of coordination between partners. The results of Experiment 1 lend support to S+, D+, and P− models of how intentional coordination affects cooperation.

### Coordination success (P+ vs. P− models)

MVL scores suggested participants coordinated equally well at both in- and anti-phase. Coordination in both of these experimental conditions was better than in the control condition. MVL scores did not significantly predict cooperation, which suggests that the social effects seen post-entrainment do not vary linearly at an individual level with coordination. This is consistent with Kirschner and Ilari (2014) and Launay et al. (2013) and rules in favor of P- style models.

MVL is a measure of coordination (i.e., the extent to which people are doing *something* together) but it is not a measure of success at performing the target coordination. For example, people trying to move in anti-phase might fail to do so and spend their time moving in-phase. MVL might still be high because the partners were coordinating, even though they had failed at the target task (see Snapp-Childs et al., 2011 and Wilson et al., 2005a for detailed analyses of this problem). A better measure of coordination for this purpose is the proportion-time-on-target. This is the proportion of time people spent coordinating at the required phase (within an error bandwidth, typically set to 20°). Proportion-time-on target, therefore, indicates how successful participants are at coordinating at the required relative phase (Wilson et al., 2010; Snapp-Childs et al., 2011, 2015). This measure was not used in our primary analysis because our control task has no target relative phase (meaning it is not possible to compute proportion-time-on-target for the control condition). However, the proportion-time-on-target can be calculated for the experimental conditions.

**Figure 3:**
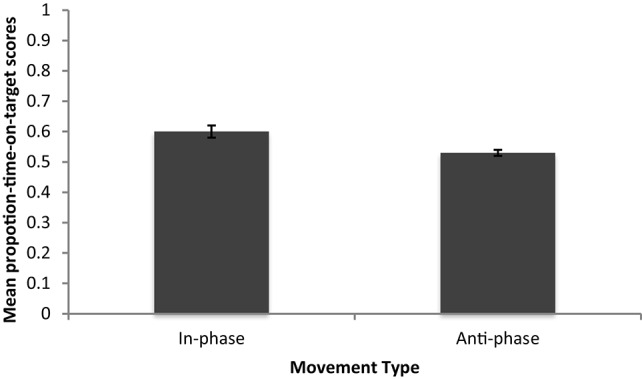
**Mean proportion-time-on-target scores for Experiment 1**.

Further analyses of the proportion-time-on-target scores revealed that those who were instructed to move in-phase were more successful than those that were instructed to move anti-phase (See Figure 3 for mean proportion-on-target-scores). Scores for those who moved at anti-phase were not normally distributed (*p* < 0.05). Because of this, an independent samples Mann-Whitney *U*-test was performed, which showed that there was a significant effect of phase on coordination (*U* = 140 *p* < 0.05), with those moving at in-phase performing significantly better than those moving anti-phase. However, coordination measured with proportion-time-on-target still did not significantly predict cooperation. A simple linear regression was run with each pair's proportion-time-on target scores and each pair's average public goods donation, to determine if coordination success predicts cooperation. A pair's coordination score did not significantly predict a pair's average cooperation score [*F*_(1, 42)_ = 0.54, *p* > 0.05, *r*^2^ = −0.011]. With the improved measure, we could identify the expected difference in performance between in- and anti-phase but the degree of coordination still did not predict the degree of cooperation. The data therefore still come down in favor of P− models; once some threshold amount of coordination has occurred, cooperation is positively affected.

### Potential mediators (D+ vs. D− models)

Against predictions, changes in trust, group cohesion and self/other overlap did not differ between conditions, suggesting that these factors do not mediate CRM's effect on cooperation (supporting D+ models). The finding that increases in group cohesion do not mediate these effects supports the work of Dong et al. (2015), Lumsden et al. (2014), and Reddish et al. (2014). However, it did not support the work of Reddish et al. (2013), Wiltermuth and Heath (2009), and Wiltermuth (2012), which found that cohesion partially mediates the relationship between CRM and its social consequences. The finding that self/other overlap does not mediate these effects contradicts studies reported by Lumsden et al. (2014) and Reddish et al. (2013).

One reason for the inconsistencies in findings could be that the present study is the first to take “before and after” coordination measures of possible mediators. It may be the case that CRM does not actually foster changes in the given variables and that previous studies simply found group differences across these variables as opposed to actual increases in mediators as a result of CRM. Alternatively it could be that the measures used here are not sensitive enough to be used as a before and after measure. Completion of the pre-test measures may have restricted participant's answers to post-test measures, therefore leaving participants unable or unwilling to give more natural responses which may have otherwise led to us finding increases in potential mediators. For the cohesion measure we saw a mean change score of 2.27 with a standard deviation of 5.63. For the overlap measure we saw a mean change score of 0.45 with a standard deviation of 1.3. Considering we find considerable variation in individual change scores, we do not believe this interpretation alone can explain our findings.

### Synchrony vs. coordination (S+ vs. S- models)

This experiment did not provide conclusive evidence that cooperation was improved by coordination more generally. Significantly greater cooperation was only seen after in-phase coordination compared to control. Anti-phase coordination did not promote greater cooperation than after control, however cooperation levels following anti-phase coordination did not significantly differ from cooperation levels following in-phase coordination either. While this might initially lend some support to the S+ class of models (synchrony, rather than coordination being required). Findings lead us to further question whether in-phase synchrony is crucial? Anti-phase coordination is a stable form of coordination (Kelso, 1995), that has been shown to affect other pro-social variables (see Cirelli et al., 2014).

The findings of Kokal et al. (2011) might shed light on the conditions necessary for different coordinations to affect pro-sociality. They provide evidence that, only when a coordination is relatively easy to perform can we attend to the social nature of the task, which is crucial to the pro-social consequences which follow. Anti-phase coordination is known to be harder and more demanding than in-phase (Kelso, 1995), as was supported by the proportion-time-on-target results in this Experiment (See Figure 3).

One potential limitation of our task was the use of simple PLDs to transmit movement information. These displays are informative about the dynamics of a person's action (Johansson, 1950; Bingham, 1987) and the success of coordinated movements in particular (Wilson and Bingham, 2008; Wilson et al., 2010) However, with their attention focused on the PLDs instead of on their partner, the social context of the coordination task might have been attenuated. In other words, using the PLD's to coordinate might dilute the social context of the coordination task.

The fact that relevant social information may be harder to detect during anti-phase coordination might explain why anti-phase coordination did not significantly differ from control. A follow up explores this possibility by having participants coordinate at both relative phases using direct visual information of each other's movements. This set up makes the social nature of the task more salient. If post task cooperation is higher following anti-phase coordination given this change, it would add further support for D− models, where an additional causally relevant factor (e.g., social context) is necessary for coordination to affect cooperation.

## Follow up 1

In this follow up, we used a modified version of the CRM task in which co-actors coordinated by looking at each other in a full-length mirror instead of using PLDs. Only the two experimental conditions (in- and anti-phase) were run in order to test whether increased social information would allow cooperation following the anti-phase condition to reach the level seen after in-phase coordination in Experiment 1. It was hypothesized that coordinating via a mirror would allow anti-phase CRM to affect cooperation similarly to in-phase CRM.

## Methods

### Participants

Forty-four psychology students at Leeds Beckett University volunteered to participate (8 males and 36 females, *M*_age_ = 19.86 year, *SD*_age_ = 1.79). All participants were naive to the aims of the study. This study was approved by the Leeds Beckett University Psychology Ethics Review Board.

### Design, measures, and procedure

The design was identical to the in-phase and anti-phase conditions from Experiment 1 except that participants watched each other using a 6 ft mirror placed horizontally 1m in front of them, below the laptop screen so that they could each view both of their upper bodies. These data were compared to the corresponding conditions from Experiment 1 to see whether enriched visual social information influenced cooperation. This follow up employed an experimental design with one between-subjects factor: Movement Phase, with two levels in- and anti-phase. This enabled us to analyse the coordination data using the superior proportion time-on-target measure. The remaining measures and procedure were identical to Experiment 1.

## Results

We first examined mood, task difficulty, task enjoyment and perceived success measures for these two new conditions to see whether these varied across conditions, using a series of Kruskal-Wallis tests (all data distributions non-normal, *p*'s < 0.05). None of these variables differed between the in-phase and anti-phase groups (all *p*'s > 0.05). It was therefore concluded that mood, task enjoyment, perceived task difficulty or perceived success did not contribute to the effects described below.

### Coordination

**Figure 4:**
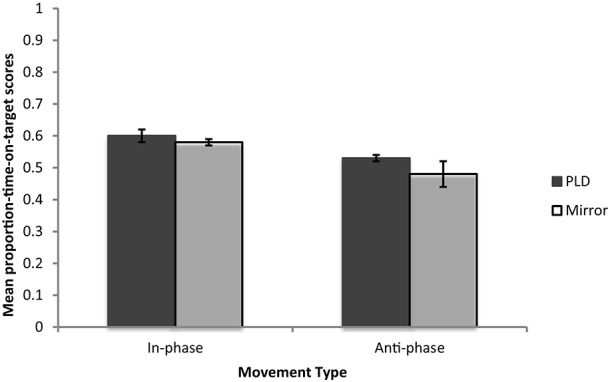
**Mean proportion-time-on-target scores for Experiment 1 and Follow up 1**.

We investigated differences in coordination scores across conditions using proportion-time-on target as a measure of coordination. The distributions of those who coordinated using the PLD and mirror at both in- and anti-phase (*p*'s < 0.05) all differed significantly from normality, and Levene's test indicated unequal variances (*F* = 15.95, *p* < 0.001). Transforming the data did not allow it to meet the normality or homogeneity assumptions. Since no non-parametric alternative to a 2-way ANOVA could be performed and Field (2013) advises that homogeneity violations are irrelevant if sample sizes amongst conditions are roughly equal (sample sizes per condition here are identical, *n* = 22), a univariate ANOVA was still used. There was only a significant effect of Movement Phase [*F*_(1, 87)_ = 14.78, *p* < 0.001], with those who moved in-phase showing greater coordination (*M* = 0.591, *SD* = 0.016) than those who moved anti-phase (*M* = 0.507, *SD* = 0.016). The effect of Coordination Information [*F*_(1, 87)_ = 2.45, *p* > 0.05] and the interaction [*F*_(1, 87)_ = 0.73, *p* > 0.05] were not significant (see Figure 4 for mean proportion-time-on-target scores). It was therefore concluded that only Movement Phase had a significant effect on coordination, with those coordinating in-phase performing more accurately than those coordinating at anti-phase. The type of available Coordination Information had no effect on coordination scores.

### Cooperation

**Figure 5:**
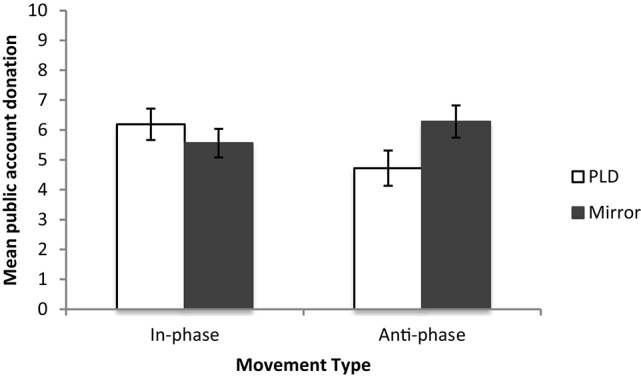
**Mean public account donations for Experiment 1 and Follow up 1**.

We then explored how rhythmically coordinating at different relative phases via differing Coordination Information affected cooperation using a 2 way ANOVA. There was no main effect of either Coordination Information or Movement Phase (*p*'s > 0.05). However, there was a significant interaction between the phase people moved at and the information they used to coordinate their movements [*F*_(1, 84)_ = 4.18, *p* < 0.05, ŋ^2^ = 0.04]. People who coordinated anti-phase via a mirror cooperated more than people who coordinated anti-phase via PLDs. There was no effect of Coordination Information on cooperation when people coordinated in-phase (see Figure 5 for the mean public account donations for each condition).

Next we conducted a simple linear regression with each pair's proportion-time-on target scores and each pair's average public goods donation, to determine if coordination success predicts cooperation. A pair's coordination score did not significantly predict a pair's average cooperation score [*F*_(1, 86)_ = 0.16, *p* > 0.05, *r*^2^ = 0.01].

### Potential mediators (group cohesion and self/other overlap)

Separate 2 Way ANOVA's were conducted for each of the potential mediators as reported in Experiment 1, no significant main effects of either Movement Phase or Coordination Information and no significant interactions were found in any of these analyses (all *p*'s > 0.05).

## Discussion

Participants coordinating at anti-phase were more cooperative if they coordinated via direct visual information of their partner's movements rather than via PLDs. In fact, those coordinating at anti-phase using the mirror saw cooperation levels comparable to participants in the in-phase condition. There was no such increase in effect for those coordinating in-phase using direct visual info. This supports the claim of Kokal et al. (2011) that the social nature of the task is an important element in why CRM has pro-social consequences (supporting a D− model), which can be obscured in more demanding tasks. This suggests that both in- and anti-phase movements are capable of affecting cooperation under the right circumstances, favoring a S− model.

Coordination scores (proportion-time-spent-on-target) again did not significantly predict cooperation scores (supporting a P− model). There is still no evidence that coordination success is driving CRM's effect on cooperation, replicating the result from Experiment 1 and supporting work by Kirschner and Ilari (2014) and Launay et al. (2013).

Greater cooperation can therefore follow either in- and anti-phase CRM compared with uncoordinated movements. However, analyses of coordination scores have shown that actual coordination does not seem to be driving this effect. The degree of coordination does not successfully predict the degree of cooperation. So what is it about the CRM task that is driving differences in cooperation? What are the critical differences between the coordinated and uncoordinated versions of this task?

## Follow up 2

In the CRM task people make the same (horizontal) movements at a shared frequency (0.75 Hz), while in the control task people make different movements (circular and vertical) at different frequencies (0.6 or 0.9 Hz). This means there are two potential differences between the CRM task and the control, type of movement and frequency of movement. Having participants perform different movements is essential to break coordination in the control task, since research shows people will end up falling into one of the two stable phases of coordination when performing the same kinds of movement unless they are trained to achieve out-of-phase coordination (Kelso, 1995).

When engaging in CRM in everyday life (e.g., when dancing), people often coordinate different movements to the same overall rhythm. What is more, Lakens (2010) has shown that people judge coordinated rhythmically moving co-actors as more entitative (seeing each other more as a unified group than as disparate individuals) regardless of whether they are coordinating exactly the same movements or not. Therefore, in order to investigate whether coordinating different movements to the same rhythm could also affect cooperation, a further follow-up condition was run in which participants coordinated different movements but to the same frequency. This is compared with the original control and the original in-phase CRM conditions from Experiment 1. It was hypothesized that coordinating different movements to the same overall frequency would foster greater cooperation than performing uncoordinated movements.

## Methods

### Participants

Twenty-two undergraduate students at Leeds Beckett University volunteered to participate (4 males and 18 females, *M*_age_ = 18.73 year, *SD*_age_ = 4.32). All participants were naive to the aims of the study. This study was approved by the Leeds Beckett University Psychology Ethics Review Board.

### Design, measures, and procedure

#### Movement task

Participants made different movements but at the same frequency (0.75 Hz). One participant moved the joystick vertically and the other in clockwise circles. Participants switched movements each trial. Otherwise the structure of the movement task was identical to the Control in Experiment 1. This condition (Coordinated) was then compared with the original in-phase (In-phase) and control condition (Control) from Experiment 1. With no defined target relative phase we analyzed coordination using MVL. The remaining measures and procedure were identical to those reported in Experiments 1.

## Results

We first examined mood, task difficulty, task enjoyment and perceived success measures to see whether these varied across conditions using a series of Kruskal-Wallis tests (All data's distributions not normal, *p*'s < 0.05). There was no significant effect of any of the above variables (all *p*'s > 0.05). It was therefore concluded that mood, task enjoyment, perceived task difficulty or perceived success did not contribute to the effects described below.

### Coordination

**Figure 6:**
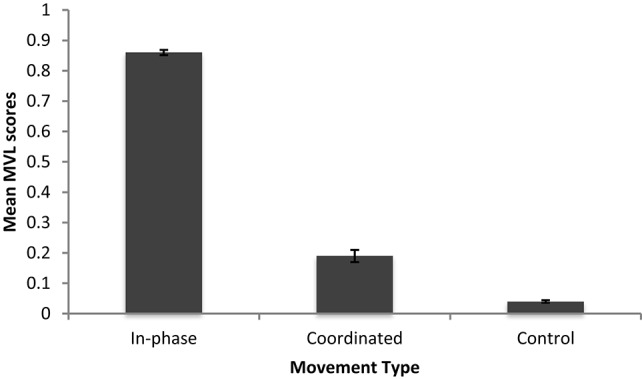
**Mean MVL scores for Experiment 1 and Follow up 2**.

We then investigated whether coordination scores differed across conditions using an independent samples Kruskal-Wallis test (recall coordination data previously failed normality tests). There was a significant effect of Movement Type on coordination scores [*H*_(2)_ = 57.83, *p* < 0.001]. Pair-wise comparisons with adjusted *p*-values showed that those who moved In-phase coordinated significantly more than those in the Coordinated condition (*U* = 3.8, *p* < 0.001) and those in the Control (*U* = 7.60, *p* < 0.001). Those in the Coordinated condition coordinated significantly more than those in the Control (*U* = 3.8, *p* < 0.001). See Figure 6 for the mean MVL scores.

### Cooperation

**Figure 7:**
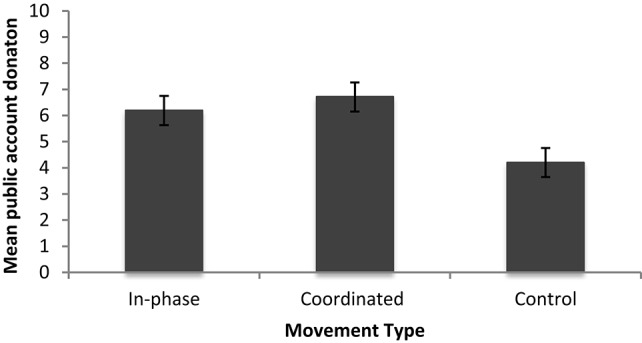
**Mean public account donations for Experiment 1 and Follow up 2**.

Next we examined the cooperation scores of those in the Coordinated compared with the original In-phase and Control conditions from Experiment 1. A univariate ANOVA was performed to see whether cooperation (mean public account donation) differed across the three movement conditions (In-phase, Coordinated and Control). There was a significant effect of Movement Type [*F*_(2, 63)_ = 5.69, *p* < 0.01 ŋ^2^ = 0.15]. Bonferroni *post-hoc* tests indicated that those who moved In-phase (*M* = 6.19, *SD* = 2.24) showed more post-coordination cooperation than those in the Control (*M* = 4.2, *SD* = 2.81, *p* < 0.05). Those in the Coordinated condition (*M* = 6.72, *SD* = 2.74) also showed more cooperation than those in the Control (*p* < 0.01). There was no difference in cooperation between those in the Coordinated condition and those who moved In-phase (*p* > 0.05). See Figure 7 for the mean public account donations for each condition.

### Potential mediators (group cohesion and self/other overlap)

A univariate ANOVA and Kruskal Wallis test (recall previous normality scores) again confirmed that there were no significant differences in any of the candidate mediators between conditions (all *p*'s > 0.05).

## Discussion

The results of this follow up show that similar levels of cooperation are seen after coordinating different movements to a common frequency as are seen after in-phase coordination, despite levels of actual coordination being significantly lower. MVL scores show that coordinating different movements to a common frequency produced significantly less tight coordination than coordinating at in-phase but significantly tighter coordination than in the original control. This was not the pattern observed in cooperation, however. The Coordinated and In-phase conditions produced comparable levels of cooperation, and both showed higher cooperation than the Control condition.

These results suggest that people do not need to perform the same type of movements for coordination to have cooperative social consequences and emphasize again that tightness of coordination is not directly linked to the magnitude of cooperation (P− model). The important factor appears to be that they coordinate to a common rhythm. Verbal reports from participants in this new condition also indicated that participants felt they were coordinating their actions. Multiple participants in this condition reported that they were trying to coordinate one full cycle of their movements to a full cycle of the other's movements (i.e., trying to complete one full up-down-up cycle on the time it took the other to complete a full circle).

This, along with the other findings reported in this paper, suggests that it is not moving at some particular phase, or a given tightness in coupling which fosters cooperation. Rather, the crucial factor appears to be just intentionally moving in time with somebody in a clearly social context, regardless of whether the same movements are performed or whether there is a specific phase locking.

## General discussion

The experiment and follow ups detailed here showed that those who perform a simple CRM task are more cooperative post-task than those who perform a control task. We also showed that similar effects obtain following anti-phase coordination and after coordinating different movements to the same overall rhythm. We found no evidence that the degree of coordination predicts the degree of cooperation, and no evidence that increases in group cohesion or blurring of self/other overlap were mediating CRM's effects on cooperation. The effects on cooperation seem to mostly stem from simply moving in time in a social context.

### Revisiting model classes

#### Synchrony (S+) vs. coordination (S−)

The results of Experiment 1 initially supported S+ models, with no significant effect of anti-phase movement on cooperation. However, the point-light displays we used only provided information about the coordinated rhythmic movement, and may detract from the social context. Increasing the salience of the social context by using mirrors led to anti-phase movements affecting cooperation to the same extent as in-phase movements. In addition, different movements at the same frequency led to greater cooperation than different movements at a different frequency. The former are still coordinated in that they are matched in time (and participants reported working to coordinate this timing). Overall, these results suggest it is temporal coordination, and not just synchrony, which can lead to pro-social consequences and so future models should be of the S− class.

#### Direct (D+) vs. indirect (D−)

Across all three studies, we found no effects of any candidate mediating variable on cooperation. It's worth noting at this point that we only looked at interactions between pairs of coordinating co-actors, and different dynamics may be at play when groups of 3 or more engage in CRM. This may be especially relevant for the group cohesion findings, as group cohesion may not be an appropriate construct for two person groups. Petersen et al. (2004) suggest group cohesion is an inter-individual attitude derived from depersonalized liking on the basis of group prototypicality. In other words, group cohesion may not be an appropriate concept for a pair of individuals. Similarly, Hogg and Turner (1985) propose that group cohesion is unlikely to be explained in terms of very personal constructs of self and other, but in terms of more general social similarities with larger numbers of people. It may be the case that group cohesion is an important factor in groups of three or more, but is not an appropriate mediator between CRM and cooperation in two person groups as is seen here.

Alternatively it may be the case that we failed to see changes in potential mediators due to a testing effect confound. It is possible that including pre as well as post-test measures of mediators may have restricted participants post-test responses. We do not however believe that this is a likely explanation, since in other work (Cross et al., Submitted) increases in group cohesion amongst larger groups have been found using these test-retest measures.

Still, results reported here showed greater cooperation amongst pairs who had performed coordinated movement than those who had performed uncoordinated movement, which was not mediated by any of the variables suggested by the literature.

We did observe an effect of social context, whereby having visual access to one's partner during the coordination task was necessary to obtain an effect of anti-phase coordination on cooperation. This pattern of results supports a D− model and is consistent with previous work showing that coordination does not have positive social consequences if the coordination task does not have a social component.

### Predicting individual (P+) or group level (P−) effects

Again, we found no evidence that the quality of coordination between participants predicted the amount of cooperation they exhibited. In addition, there was no increase in coordination stability in anti-phase movements when co-actors coordinated via direct movement information, but cooperation did increase. Once people perceive that they are temporally coordinating in a social context, greater cooperation follows. This supports P- class models for future work.

### Limitations

The findings presented in this paper apply only to cases of intentional coordination. They may not necessarily generalize to instances of unintentional coordination. This remains an interesting point for future work to explore. A further limitation is that the results of Experiment 1 were analyzed in conjunction with both of the follow ups. These results are effectively exploratory and require independent replication.

## Summary

The current studies demonstrated that people who engage in a simple CRM task are more cooperative post task than people who engage in a control task. By relying on a well-defined and well-understood CRM task (see Golonka and Wilson, 2012 for a review), we were able to systematically manipulate a variety of task-critical parameters. This level of control means that we were able to begin identifying properties that eventual explanatory models of CRMs effect on cooperation must possess. In summary, our results indicate that this effect (1) follows from coordination generally, not just in-phase synchrony, (2) is indirect, in that coordination must occur in a social context; but direct in that the effect does not depend on coordination causing changes in mediating variables, and (3) is not proportional to individual level coordination performance.

## Ethics statement

Participants were provided with an information sheet explaining the nature of the research prior to making an appointment to participate in the research. When potential participants arrived at their appointment, they were provided with a detailed consent form detailing their rights as participants. They were free to ask the researcher any questions. If they were happy to continue, they signed the consent form and the experimental session began. The research was approved by the Leeds Beckett University Psychology Ethics Committee

## Author contributions

LC conducted the experiments and analyses reported in this paper as part of his Ph.D. under the supervision of SG (director of studies) and AW (second supervisor). All authors therefore contributed to the design and analysis of the studies and we all contributed equally to the writing.

### Conflict of interest statement

The authors declare that the research was conducted in the absence of any commercial or financial relationships that could be construed as a potential conflict of interest.

## Footnotes

^1^Anti-phase is typically less stable than in-phase; this is one of the hallmarks of coordinated rhythmic movement. The lack of a difference here is a common issue with the MVL measure because it does not account for what relative phase people are actually performing. Anti-phase coordination can show an elevated MVL if people end up switching to in-phase coordination, and do that well (Wilson et al., 2005a,b; Snapp-Childs et al., 2011). We address this in detail in the Discussion section.
