# Supplementary material for: How Moving Together Brings Us Together: When Coordinated Rhythmic Movement Affects Cooperation
Source: Front Psychol. 2016 Dec 22;7:1983. doi: 10.3389/fpsyg.2016.01983 (PMC5177969; doi:10.3389/fpsyg.2016.01983)

Appendix 1. The measure of self/other overlap.

Please choose the picture that best describes how you feel right now.

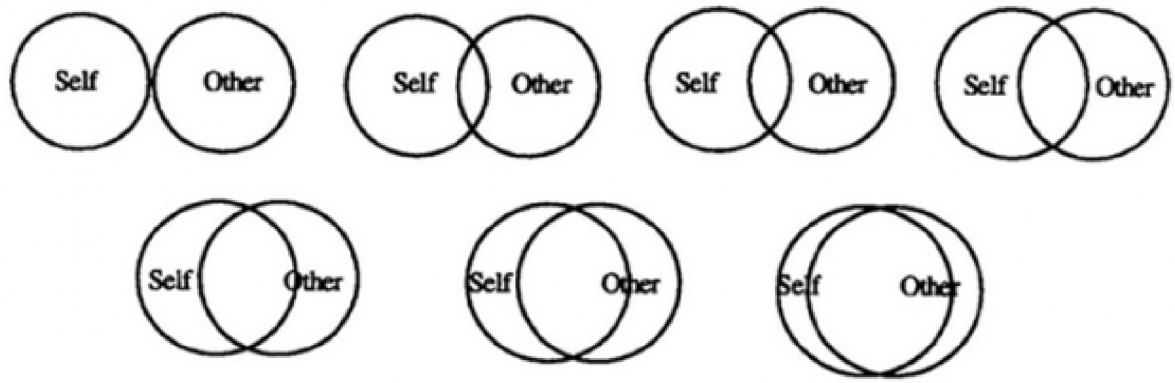

Supplement: Supplementary file 1 [file DataSheet1.pdf]
