# Supplementary material for: How Moving Together Brings Us Together: When Coordinated Rhythmic Movement Affects Cooperation
Source: Front Psychol. 2016 Dec 22;7:1983. doi: 10.3389/fpsyg.2016.01983 (PMC5177969; doi:10.3389/fpsyg.2016.01983)
Supplement: Supplementary file 2 [file DataSheet2.pdf]

Appendix 2. The cohesion scale.

Please record your responses to the following questions by placing a | at the appropriate place on the continuum. Please do so as carefully and neatly as possible.

Very unhappy | ***How happy do you feel right now?*** | Very happy

{---------------------------------------------------------------------------------------}

Not at all close | ***How close do you feel to the other participants?*** | Very close

{---------------------------------------------------------------------------------------}

Not at all similar | ***How similar do you feel to the other participants?*** | Very similar

{---------------------------------------------------------------------------------------}

Not at all. | ***How connected do you feel to the other participant?*** | Very much so

{---------------------------------------------------------------------------------------}

Not at all. | ***How much do you trust the other participants?*** | Very much so

{---------------------------------------------------------------------------------------}
