# Supplementary material for: How Moving Together Brings Us Together: When Coordinated Rhythmic Movement Affects Cooperation
Source: Front Psychol. 2016 Dec 22;7:1983. doi: 10.3389/fpsyg.2016.01983 (PMC5177969; doi:10.3389/fpsyg.2016.01983)
Supplement: Supplementary file 3 [file DataSheet3.pdf]

Appendix 3. The economic game

Each round you have ten imaginary tokens. Which you must divide between two accounts, a private account and a public account. You can do this in any way you like, you can put all your tokens in one account and none in the other, or split them however you wish.

At the end of the game your Tokens will be tallied, for each token in your private account you will receive five points, for each token in the public accounts each player will receive three points

You will then have the opportunity to try an increase your total number of **Whoever collects the most points overall will win £40 worth of Amazon vouchers**.

Round Number	Private Account	Public Account
1		
2		
3		
4		
5		

You have the option of investing some of the points you have already earned with the other player. Any investments you make will automatically be doubled (so 10 points would double to 20 points), however, it is then up to the other player how many of these points they then transfer back to you and how many they keep for themselves.

Equally the other person has the option of investing a number of points with you and you will be deciding how many of these points to transfer back.

As the investor you can choose to transfer no points, a quarter of your points half of your points or all of your points to the other player. Once transferred, these points will automatically double but it is then up the other player how many of these points to return to you.

I wish to invest the following number of points with the other player

Please circle one of the following options ……

None ***a quarter*** ***half*** ***all***

As the investee, you can choose how many of the other player's transferred points you wish to return to them. The points returned will be added to the other players total and the points that are not returned will be added to your own total.

I wish to return the following number of points to the other player

Please circle one of the following options ……

The original investment plus all of the bonus earned.

The original investment plus half of the bonus earned.

Only the original investment.

No points at all.
